# Association of Serum Ferritin and Kidney Function with Age-Related Macular Degeneration in the General Population

**DOI:** 10.1371/journal.pone.0153624

**Published:** 2016-04-20

**Authors:** Il Hwan Oh, Eun Young Choi, Joon-Sung Park, Chang Hwa Lee

**Affiliations:** Department of Internal Medicine, Hanyang University College of Medicine, Seoul, Korea; The Pennsylvania State University Hershey Medical Center, UNITED STATES

## Abstract

Ferritin is considered to be a marker of the body’s iron stores and has a potential relationship with the systemic manifestations of inflammatory reactions. Data on the association between increased levels of serum ferritin and ocular problems are limited, particularly in relation to age-related macular degeneration (AMD). Serum ferritin levels, as a possible clinical parameter for predicting AMD, were analyzed in anthropometric, biochemical, and ophthalmologic data from a nation-wide, population-based, case-control study (KNHNES IV and V). All native Koreans aged ≥ 20 years and who had no medical illness were eligible to participate. Among them, 2.9% had AMD, and its prevalence was found to increase in the higher ferritin quintile groups (P_trend_ < 0.0001). In multiple linear regression analysis, serum ferritin level was closely related to conventional risk factors for AMD. Comparison of early AMD with a control group showed that serum ferritin levels were closely associated with AMD (OR = 1.004, 95% *CI* = 1.002–1.006), and further adjustment for age, gender, serum iron, and kidney function did not reduce this association (OR = 1.003, 95% *CI* = 1.001–1.006). Furthermore, the relationship between ferritin quintile and early AMD was dose-dependent. Thus, an increased level of serum ferritin in a healthy person may be a useful indicator of neurodegenerative change in the macula. A large population-based prospective clinical study is needed to confirm these findings.

## Introduction

Age-related macular degeneration (AMD) is a neurodegenerative change in the macula at the center of the retina that is closely related to loss of detailed central vision [1]. Population growth, obesity, and widespread adoption of a ‘western type’ of diet may be contributing to the increase of AMD in older people, which is now a worldwide health problem [1,2]. Because of its progressive nature, early identification of possible risk factor(s) is critical for preventing the resulting disability.

Macular degeneration is a typical multifactorial disease [2], and complicated interactions between aging, genetic factors, environmental influences, lifestyle changes, and underlying diseases can influence its development and severity [2–4]. Epidemiologic studies show that several chronic diseases such as metabolic syndrome (MetS), characterized by subclinical systemic inflammation, are strongly associated with AMD [5]. In the line of evidences, several experimental studies reveal that oxidative stress-induced inflammatory reactions may play a role in the initiation and progression of AMD [6–8]. Such evidences suggest that increased pro-inflammatory hormones or cytokines can contribute to the development of AMD and that this disease may be a neuronal manifestation of systemic inflammatory responses. However, there is little clinical evidence supporting such association.

Iron is an essential mineral for all living cells and participates in a wide array of physiologic functions [9,10]. However, shortage of iron-sequestrating proteins, including ferritin, and inappropriate accumulation of this transition metal are closely related to the generation of cytotoxic reactive oxygen species, which results in initiation and progression of various non-specific inflammatory responses and neurodegenerative diseases [10–12]. On the other hand, the increased appearance of ferritin in extracellular spaces may be the result of extensive cell damage, and the subsequent breakdown of ferritin could contribute to increased release of sequestrated iron into the blood stream [13]. Moreover, recent reports suggest that ferritin itself is cytotoxic and may inhibit growth in neighboring cells [13,14]. Furthermore, an increase in serum ferritin is closely related to ocular manifestation of systemic inflammatory processes [15]. However, little is known about the association of serum ferritin with macular degeneration. In this study, we aimed to determine whether increased levels of serum ferritin are related to the development of AMD.

## Methods

### Study population

Data were collected from public-use data sets in the Korean National Health and Nutrition Examination Survey (KNHANES) conducted by the Korea Centers for Disease Control and Prevention (KCDC) among non-institutionalized Korean civilians between 2008 and 2012. All participants in our study were volunteers and all provided written informed consent before enrollment. Their records, excluding survey date and home address, were anonymized prior to analysis. The study was approved by the Institutional Review Board (IRB) of the KCDC (IRB: 2008-04EXP-01-C, 2009-01CON-03-2C, 2010-02CON-21-C, 2011-02CON-06-C, 2012-01EXP-01-2C).

**Fig 1:**
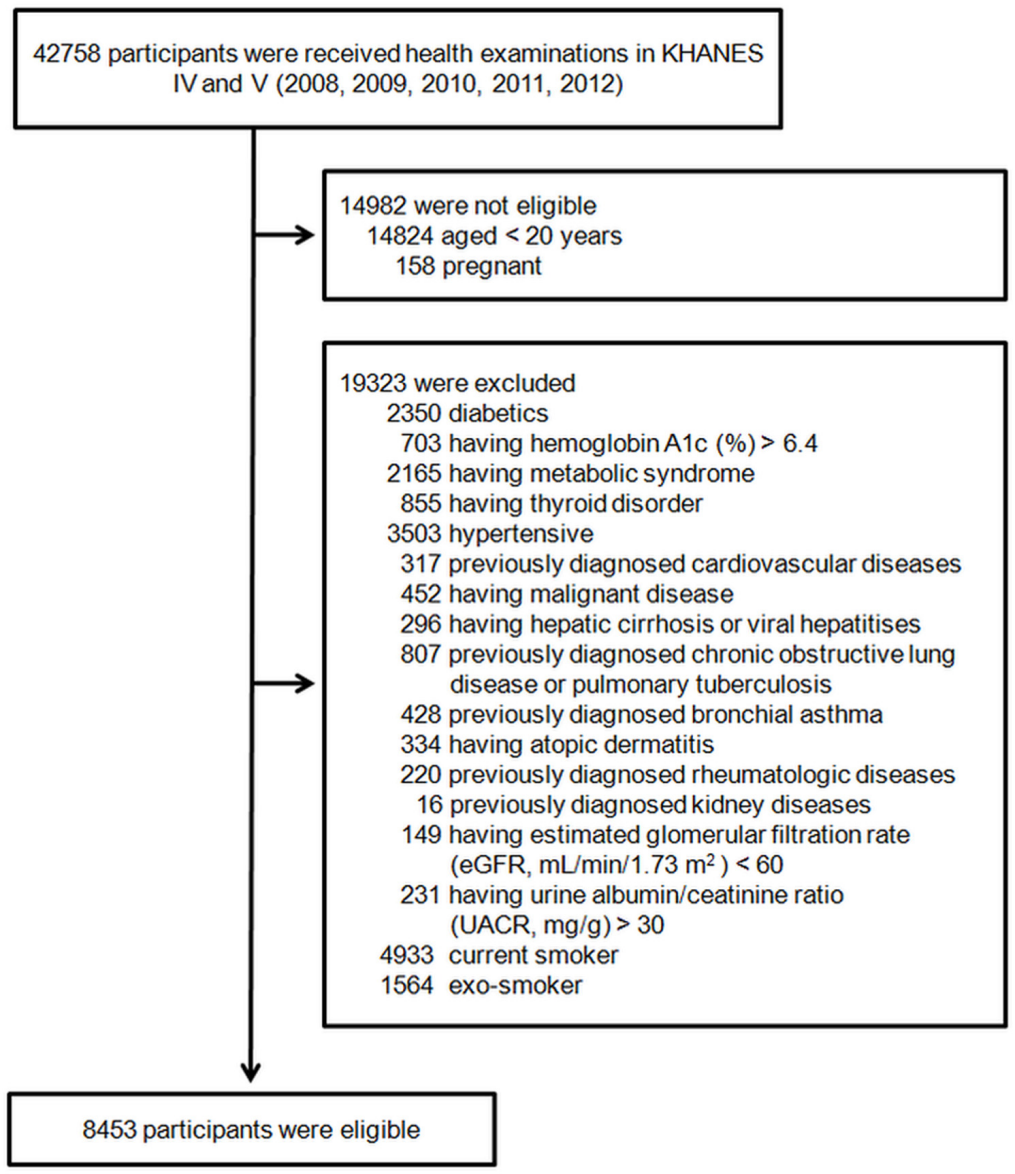
Flow chart of the study enrollment process. KHANES, The Korean National Health and Nutritional Examination Survey.

A total of 42758 individuals were participated in the KHANES 2008–2012. Individuals were excluded from the present analysis for any of the following reasons: incomplete data (anthropometric data or laboratory data), < 20 years of age, history of smoking, and pregnancy. Those with a history of medical problems or with estimated glomerular filtration rate (eGFR) < 60 mL·min^-1^·1.73 m^-2^ or urine albumin/creatinine ratio (UACR) > 30 mg·g^-1^ creatinine were also excluded (Fig 1). The final 8453 participants were divided into two groups according to their AMD examination results.

### Anthropometric and clinical measurements

Anthropometric measurements were made by well-trained examiners. Participants wore a lightweight gown or underwear. Height was measured to the nearest 0.1 cm using a portable stadiometer (Seriter, Bismarck, ND). Weight was measured to the nearest 0.1 kg on a calibrated balance-beam scale (Giant-150N; Hana, Seoul, Korea).

Waist circumference was measured using a flexible tape at the narrowest point between the lowest border of the rib cage and the uppermost lateral border of the iliac crest at the end of normal expiration. Body mass index was calculated as weight in kilograms divided by square of the height in meters. The conicity index was computed from the weight, height, and waist circumference [16].

Blood pressure (BP) was measured three times, using a mercury sphygmomanometer (Baumanometer; Baum, Copiague, NY) while subjects were in a sitting position following a 5-minute rest period. The average values of the three recorded systolic and diastolic BPs were used in the analysis.

### Laboratory tests

Venous blood samples were collected after 8 h overnight fasting. Serum ferritin was measured with an immunoradiometric assay using a 1470 WIZARD^®^ automatic gamma counter (PerkinElmer, Waltham, MA, USA) in the central laboratory. Fasting plasma concentrations of glucose and lipid were measured enzymatically using a Hitachi Automatic Analyzer 7600 (Hitachi, Tokyo, Japan). Glycated hemoglobin levels were determined by high-performance liquid chromatography with an automated HLC-723G7 analyzer (Tosoh Corporation, Tokyo, Japan). Serum creatinine levels were measured colorimetrically (Hitachi Automatic Analyzer 7600) and eGFR was calculated using the Chronic Kidney Disease Epidemiology Collaboration (CKD-EPI) equation [17]. To obtain the UACR, urinary albumin was measured in spot urine using the immunoturbidimetric method and urinary creatinine was measured using the colorimetric method.

### Assessment of AMD

AMD examinations were made by ophthalmologists from the Korean Ophthalmological Society, in cooperation with KCDC, and were trained by the National Epidemiological Survey Committee of the Korean Ophthalmological Society. A non-mydriatic fundus camera (TRC-NW6S, Topcon, Tokyo, Japan) was used by ophthalmologists or ophthalmologists-in-training to obtain fundus photographs. Early AMD was diagnosed in a fundus photograph if one of two conditions was present: (1) soft indistinct drusen or reticular drusen or (2) hard or soft distinct drusen with pigment abnormality but without signs of late AMD [18]. Late AMD was defined as the presence of wet or dry (geographic atrophy) AMD [18]. Wet AMD was defined as detachment of the retinal pigment epithelium (RPE) or neurosensory retina, the presence of hemorrhages in the subretinal or sub-RPE space, or a disc-form scar in the macular area [18]. Dry AMD was defined as a circular, discrete depigmented area ≥ 175 μm diameter with visible choroidal vessels [18].

### Statistical analysis

All data, including socio-demographic data, medical conditions, anthropometric and clinical measurements, and laboratory and ophthalmologic results, are presented as means ± SE or frequencies (and proportions). Data were analyzed using sampling weights to account for multistage and stratified sampling. The *t*-test was used to compare quantitative variables and Pearson’s chi-square test to compare proportions for categorical variables. The Cochran-Armitage test was used to evaluate trends in prevalence of ocular disease across the serum ferritin quintiles. Odds ratios (ORs) with 95% confidence intervals (CIs) were calculated in multiple logistic regression models according to the presence of macular degeneration (control vs. case). A two-tailed P < 0.05 was considered statistically significant. Statistical Analysis Software version 9.3 (SAS Institute Inc, Cary, NC, USA) was used for all analyses.

## Results

### Baseline characteristics

The participants (n = 8453) comprised 1372 men and 7081 women with a mean age 45.4 ± 13.8 years, and they were divided into two groups according to degenerative changes of the macula: the control group (n = 8206) and the AMD group (n = 247). Participants with AMD were older, had higher serum ferritin levels, and were more exposed to sunlight than those without AMD. Those in the AMD group were more likely to be hypertensive and obese, with worse lipid profiles and poorer kidney function. There were no differences between the groups in other baseline characteristics (Table 1).

**Table 1:** General characteristics of age-related macular degeneration (AMD).

Variables	Control group (n = 8206)	AMD group (n = 246)	*P*
Age (years)	41.8 ± 0.2	61.2 ± 1.1	<0.0001
Gender (% male)	1336 (16)	36 (15)	0.1801
Systolic BP (mmHg)	112.2 ± 0.2	122.5 ± 1.4	<0.0001
Diastolic BP (mmHg)	74.1 ± 0.2	75.9 ± 0.2	0.0018
Body mass index (kg/m^2^)	23.00 ± 0.05	22.84 ± 0.23	0.4852
Waist circumference (cm)	77.5 ± 0.1	78.7 ± 0.6	0.0523
Conicity index (m^1½^·kg^-½^)	1.168 ± 0.001	1.211 ± 0.006	<0.0001
eGFR (mL·min^-1^·1.73 m^-2^)	99.6 ± 0.2	90.5 ± 1.0	<0.0001
Hemoglobin (g/dL)	13.46 ± 0.02	13.37 ± 0.11	0.4639
Iron (μg/dL)	106.5 ± 0.9	100.0 ± 3.6	0.0800
TIBC (μg/dL)	320.7 ± 1.0	304.7 ± 3.5	<0.0001
Ferritin (ng/mL)	55.2 ± 0.2	75.1 ± 6.7	0.0032
Glucose (mg/dL)	91.0 ± 0.1	92.6 ± 0.7	0.0254
Hemoglobin A1c (%)	5.48 ± 0.01	5.63 ± 0.03	<0.0001
Triglyceride (mg/dL)	105.6 ± 1.1	120.1 ± 6.4	0.0238
HDL-cholesterol (mg/dL)	54.2 ± 0.2	54.1 ± 1.9	0.9507
LDL-cholesterol (mg/dL)	112.7 ± 0.9	123.8 ± 5.7	0.0562
Intact PTH (pg/mL)	64.5 ± 0.7	64.8 ± 3.0	0.9012
UACR (mg/g)	4.0 ± 0.1	4.7 ± 0.5	0.1819
Sunlight exposure (h/day)			0.0008
<2	3243 (40)	104 (42)	
2–5	945 (12)	37 (15)	
>5	390 (5)	36 (15)	
Dietary iron intake (mg/day)	38.4 ± 0.5	29.5 ± 2.2	<0.0001
Dietary fat intake (g/day)	13.9 ± 0.2	14.7 ± 1.0	0.4415

Results are expressed as mean ± SE or frequencies (and proportions). eGFR, estimated glomerular filtration rate; TIBC, total iron binding capacity; BP, blood pressure; LDL, low-density lipoprotein; PTH, parathyroid hormone; UACR, urine albumin/creatinine ratio.

### Serum ferritin

Crude and adjusted linear regression analysis was used to discover possible relationships between serum ferritin and other baseline characteristics associated with AMD (Table 2). In the adjusted analysis, serum ferritin was positively correlated with diastolic blood pressure, central obesity, and triglyceride level. There was an inverse correlation between serum ferritin and HDL-cholesterol level. Serum ferritin was not related to daily intake of iron or fat in the adjusted analysis.

**Table 2:** Multivariate linear regression analysis of the relation of serum ferritin level to other characteristics.

	Crude		Model I	
Variables	β	*P*	β	*P*
Age (years)	0.3140	<0.0001		
Systolic BP (mmHg)	0.6938	<0.0001	0.0739	0.1540
Diastolic BP (mmHg)	1.2437	<0.0001	0.2474	0.0018
Body mass index (kg/m^2^)	2.9574	<0.0001	1.2731	<0.0001
Waist circumference (cm)	1.7180	<0.0001	0.6071	<0.0001
Conicity index (m^1½^·kg^-½^)	135.18	<0.0001	65.698	<0.0001
eGFR (mL·min^-1^·1.73 m^-2^)	-0.6718	<0.0001	-0.0656	0.2981
Hemoglobin (g/dL)	18.559	<0.0001	9.9402	<0.0001
Iron (μg/dL)	0.2850	<0.0001	0.1588	<0.0001
TIBC (μg/dL)	-0.4618	<0.0001	-0.3385	<0.0001
Glucose (mg/dL)	0.8139	<0.0001	0.3390	0.0002
Hemoglobin A1c (%)	1.3262	0.7609		
Triglyceride (mg/dL)	0.1372	<0.0001	0.0632	<0.0001
HDL-cholesterol (mg/dL)	-0.6315	<0.0001	-0.1263	0.0437
LDL-cholesterol (mg/dL)	0.1077	0.0505		
Intact PTH (pg/mL)	-0.0898	0.0547		
UACR (mg/g)	-0.1389	0.6478		
Dietary iron intake (mg/day)	0.4015	0.0026	0.0078	0.8953
Dietary fat intake (g/day)	0.1720	<0.0001	-0.0012	0.9667

Model I: adjusted for age and gender.

### Association of serum ferritin with AMD

**Fig 2:**
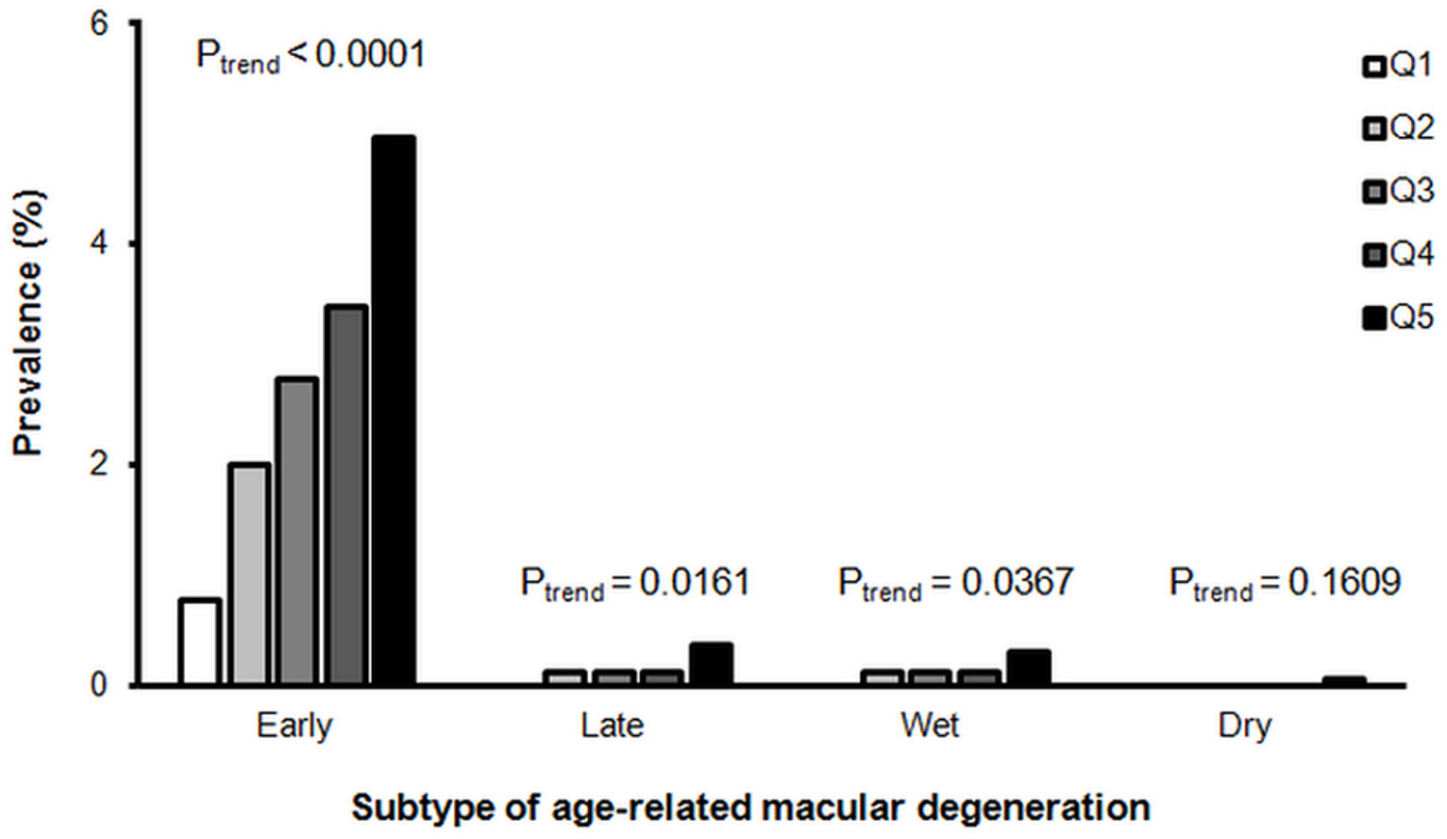
Prevalence of age-related macular degeneration subtype in relation to serum ferritin quintile (Q). *calculated using the Cochranaaaaaaaaaa-Armitage test for trend.

To assess the dependence of AMD on serum ferritin level, all participants were divided into quintiles according to gender and serum ferritin level. Early AMD was the more common subtype, and its prevalence increased stepwise in ascending quintiles 1 to 5 (P_trend_ < 0.0001) (Fig 2). Late AMD was prominent only in quintile 5.

Logistic regression analysis was used to determine factors associated with visual disturbance (Table 3). When participants with early AMD were compared to controls, the crude OR of serum ferritin for AMD was 1.004 (95% *CI* = 1.002–1.006). Further adjustment for age, gender, serum iron, and kidney function did not attenuate this association (adjusted OR = 1.003, 95% *CI* = 1.001–1.006).

**Table 3:** Multivariate logistic regression for early AMD.

	Crude		Model II	
Variable	OR	95% *CI*	OR	95% *CI*
Age (years)	1.093	1.082–1.105		
Gender (vs. male)	1.466	0.905–2.375		
Systolic BP (mmHg)	1.035	1.027–1.043	1.007	0.996–1.018
Diastolic BP (mmHg)	1.017	1.002–1.032	1.011	0.990–1.031
Body mass index (kg/m^2^)	0.983	0.936–1.032		
Waist circumference (cm)	1.012	0.997–1.028		
Conicity index (m^1½^·kg^-½^)	720.1	126.6–999.9	3.236	0.025–3.769
eGFR (mL·min^-1^·1.73 m^-2^)	0.958	0.950–0.966		
Hemoglobin (g/dL)	0.956	0.867–1.056		
Iron (μg/dL)	0.996	0.993–1.000		
TIBC (μg/dL)	0.992	0.988–0.996	0.995	0.991–1.000
Ferritin (ng/dL)	1.004	1.002–1.006	1.003	1.001–1.006
Glucose (mg/dL)	1.018	1.004–1.032	1.016	0.964–1.005
Hemoglobin A1c (%)	3.884	2.196–6.868	1.024	0.524–2.001
Triglyceride (mg/dL)	1.002	1.001–1.003	1.002	0.994–1.001
HDL-cholesterol (mg/dL)	0.999	0.978–1.029		
LDL-cholesterol (mg/dL)	1.011	1.001–1.022	1.002	0.987–1.016

Model II: adjusted for age, gender, serum iron, and kidney function. OR, odd ratio; *CI*, confidence interval.

**Fig 3:**
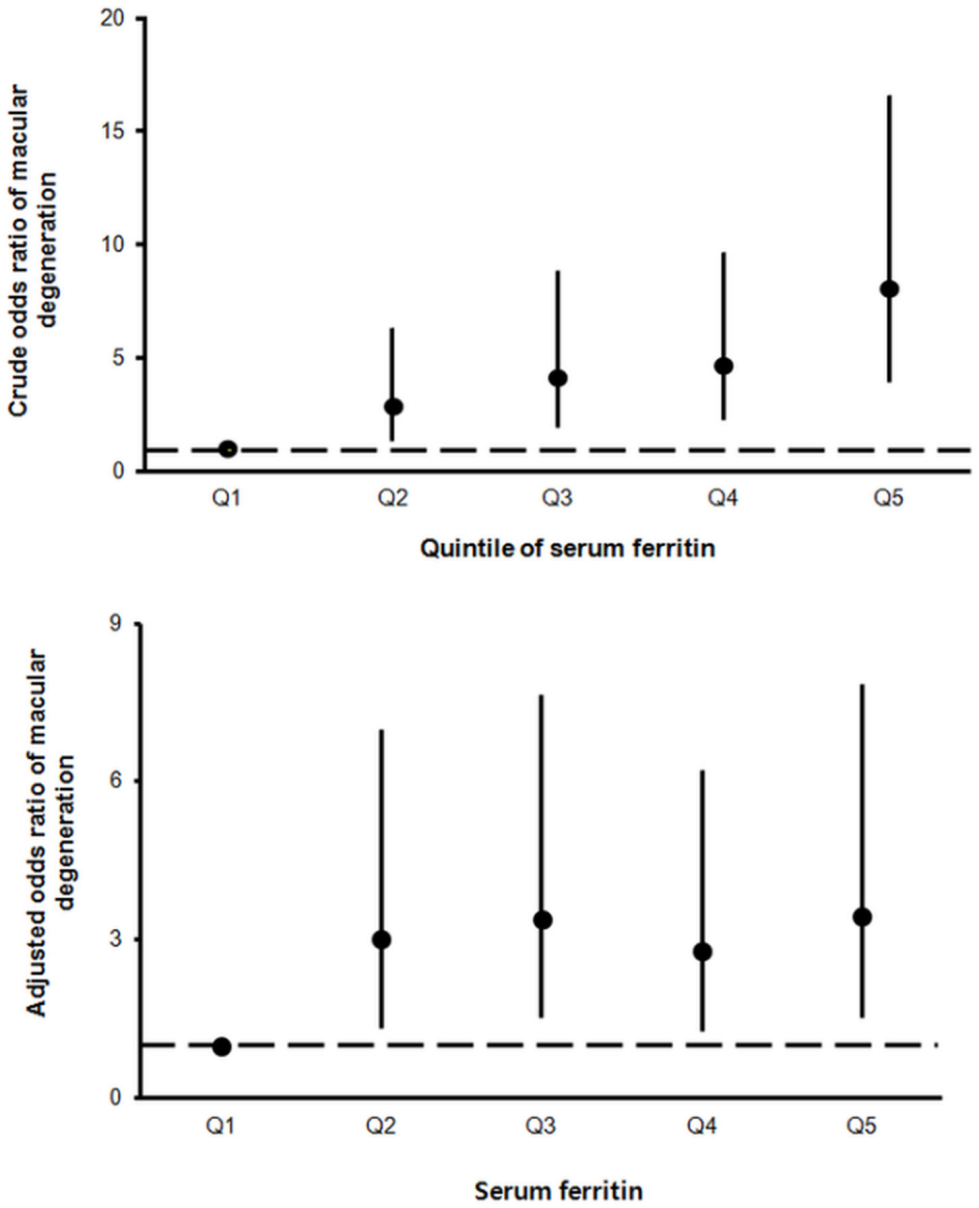
Risk of age-related macular degeneration in relation to serum ferritin quintile. (A) Crude odds ratios (ORs) and 95% confidence intervals for AMD as a function of serum ferritin quintile (Q), using Q1 as the reference. (B) After adjustment for age, gender, serum iron, and kidney function, participants in Q5 had a significantly greater risk of early AMD than those in Q1 (as the reference).

Fig 3 shows the ORs of the serum ferritin quintiles in relation to early AMD. Using the lowest quintile as reference, the crude ORs of AMD were: 2.771 (95% *CI* = 1.253–6.125) for quintile 2; 4.088 (95% *CI* = 1.920–8.704) for quintile 3; 4.833 (95% *CI* = 2.313–10.102) for quintile 4; 7.242 (95% *CI* = 3.519–14.904) for quintile 5. After adjustment for age, gender, and kidney function, the adjusted OR of early AMD were: 1.483 (95% *CI* = 0.910–2.418) for quintile 2; 1.411 (95% *CI* = 0.892–2.234) for quintile 3; 1.403 (95% *CI* = 0.925–2.127) for quintile 4; 1.719 (95% *CI* = 1.101–2.683) for quintile 5.

## Discussion

Our results show that increase of serum ferritin is strongly related to the metabolic risk factors for macular degeneration, and that there is stepwise increase in risk of AMD across serum ferritin quintiles in the general population. These findings suggest that serum ferritin could be of value in predicting ocular problems.

Study participants with AMD had higher serum ferritin levels than the control group, even below the diagnostic criteria for hyperferritinemia, and serum ferritin levels were strongly related to each component of MetS. Our results are consistent with other reports showing that MetS and its components are closely associated not only with the development of AMD, but also with increased levels of serum ferritin [5, 19–24]. Ferritin is an intracellular protein recognized for its iron sequestration and storage ability and its participation in physiologic and pathologic processes that depend on iron availability [25,26]. In disease conditions, increased oxidative stress or pro- and anti-inflammatory cytokines may induce over-production of ferritin and its subtypes, which can have further toxic effects on neighboring cells and tissues [25]. Thus, ferritin may play an intermediate role between systemic inflammation and its local manifestations, and a mild increase in serum ferritin could indicate initiation of inflammatory and degenerative disease processes.

Our logistic regression analysis showed that the components of MetS are not independently associated with early AMD. Data on the association between MetS components and development of ocular complications, especially early AMD, are conflicting. Recent epidemiologic studies showed that MetS and its components were possibly associated with progression to late AMD but not with early AMD [5, 27]. In the present analysis aiming to determine the exact connection between conventional risk factors and early AMD, individuals with diabetes, hypertension, or MetS were excluded to avoid the possible confounding effect of metabolic stress. We found that serum ferritin was more strongly associated with early AMD than MetS or its components. Furthermore, we also used an adjustment method to explore possible interactions of age, gender, serum iron, and kidney function with serum ferritin level in the development of AMD. Finally, our multiple logistic model demonstrated that serum ferritin was an independent predictor of early AMD, indicating that serum ferritin may be more sensitive than MetS components in predicting the risk of early AMD.

Our subgroup analysis showed a dose-dependent relationship between serum ferritin and risk of early AMD. The retina is made of nervous tissue and aberrant iron metabolism has the potential to contribute to degenerative change in the retina via oxidative injuries [28]. One study for iron accumulation in AMD retinas proposed that excess iron may play a role in the early pathogenesis of AMD via iron-related oxidative stress, suggesting serum ferritin may reflect altered iron homeostasis in the retinas [29]. On the other hand, serum ferritin has been widely accepted as an acute-phase reactant and is nonspecifically elevated in a wide variety of systemic inflammatory states [30]. In health, cytoplasmic ferritin is protective through its ability to chelate free iron [15]. In pathologic states, extracellular ferritin released from damaged cells can be a source of iron in an unliganded form; this may create hydroxyl radicals and consequent further cellular damage [13]. Thus, our results provide clinical evidence that increased serum ferritin, as a biomarker of the body’s iron stores and an inflammatory reaction in the ocular system, enhances the harmful effect of various insults on the development of early AMD.

There are several limitations to our study. First, considering that inflammation contribute to the development of AMD, it would have been informative to include various inflammatory markers in our study. Regrettably, data on other inflammatory biomarkers such as C-reactive protein and interleukins were not analyzed because they were not measured in the study; therefore, any relationship between pro-inflammatory mediators and AMD development could not be determined. However, the association between CRP and serum ferritin is widely known [31], and serum ferritin could reflect not only iron storage but also inflammation in the body. Hence, serum ferritin may be a preferred predictor for the risk of AMD regardless of other inflammatory markers. Second, the development of AMD may have been influenced by a wide variety of factors. Because of the limitations of cross-sectional studies, we could not adjust for many factors other than age, gender, serum iron, and daily intake of iron or fat. Third, because of the self-reporting of medical history, medication, and use of tobacco and alcohol, a social-desirability bias could not be ruled out and may have been responsible for results and conclusions that conflicted with previous research. Finally, participants might have forgotten pertinent relevant details.

The results of our study suggest that a relatively high level of serum ferritin in the healthy population is associated with progression of AMD and may be an important predictor of AMD, especially the early subtype of AMD. To the best of our knowledge, this study is the first to define the relationship between serum ferritin and early AMD in the general population. A large population-based prospective clinical study is needed to identify the mechanisms involved and the clinical implications.

## References

[1] Cho E, Hung S, Willett WC, Spiegelman D, Rimm EB, Seddon JM, et al Prospective study of dietary fat and the risk of age-related macular degeneration. Am J Clin Nutr. 2001;73: 209–218. 11157315 10.1093/ajcn/73.2.209

[2] Nowak JZ. Age-related macular degeneration (AMD): pathogenesis and therapy. Pharmacol Rep. 2006;58:353–363. 16845209

[3] Park SJ, Lee JH, Woo SJ, Ahn J, Shin JP, Song SJ, et al Age-related macular degeneration: prevalence and risk factors from Korean National Health and Nutrition Examination Survey, 2008 through 2011. Ophthalmology. 2014;121: 1756–1765. 10.1016/j.ophtha.2014.03.022 24813632

[4] Aoki A, Tan X, Yamagishi R, Shinkai S, Obata R, Miyaji T, et al Risk Factors for Age-Related Macular Degeneration in an Elderly Japanese Population: The Hatoyama Study. Invest Ophthalmol Vis Sci. 2015 3 18 pii: IOVS-14-16339. 10.1167/iovs.14-16339 25788651

[5] Maralani HG, Tai BC, Wong TY, Tai ES, Li J, Wang JJ, et al Metabolic syndrome and risk of age-related macular degeneration. Retina. 2015;35: 459–466. 10.1097/IAE.0000000000000338 25207946

[6] Lederman M, Hagbi-Levi S, Grunin M, Obolensky A, Berenshtein E, Banin E, et al Degeneration modulates retinal response to transient exogenous oxidative injury. PLoS One. 2014 2 21;9(2):e87751 10.1371/journal.pone.0087751 24586289 PMC3931611

[7] Kauppinen A, Niskanen H, Suuronen T, Kinnunen K, Salminen A, Kaarniranta K. Oxidative stress activates NLRP3 inflammasomes in ARPE-19 cells—implications for age-related macular degeneration (AMD). Immunol Lett. 2012;147: 29–33. 10.1016/j.imlet.2012.05.005 22698681

[8] Gao J, Liu RT, Cao S, Cui JZ, Wang A, To E, et al NLRP3 inflammasome: activation and regulation in age-related macular degeneration. Mediators Inflamm. 2015;2015:690243 10.1155/2015/690243 25698849 PMC4324923

[9] Richer S, Rudy D, Statkute L, Karofty K, Frankowski J. Serum iron, transferrin saturation, ferritin, and dietary data in age-related macular degeneration. Am J Ther. 2002;9: 25–28. 11782816 10.1097/00045391-200201000-00006

[10] Schipper HM. Neurodegeneration with brain iron accumulation—clinical syndromes and neuroimaging. Biochim Biophys Acta. 2012;1822: 350–360. 10.1016/j.bbadis.2011.06.016 21782937

[11] Loh A, Hadziahmetovic M, Dunaief JL. Iron homeostasis and eye disease. Biochim Biophys Acta. 2009;1790:637–649. 10.1016/j.bbagen.2008.11.001 19059309 PMC2718721

[12] Batista-Nascimento L, Pimentel C, Menezes RA, Rodrigues-Pousada C. Iron and neurodegeneration: from cellular homeostasis to disease. Oxid Med Cell Longev. 2012;2012:128647 10.1155/2012/128647 22701145 PMC3369498

[13] Kell DB, Pretorius E. Serum ferritin is an important inflammatory disease marker, as it is mainly a leakage product from damaged cells. Metallomics. 2014;6: 748–773. 10.1039/c3mt00347g 24549403

[14] Krenn MA, Schürz M, Teufl B, Uchida K, Eckl PM, Bresgen N. Ferritin-stimulated lipid peroxidation, lysosomal leak, and macroautophagy promote lysosomal "metastability" in primary hepatocytes determining in vitro cell survival. Free Radic Biol Med. 2015;80: 48–58. 10.1016/j.freeradbiomed.2014.12.007 25532933

[15] Lin SC, Wang SY, Yoo C, Singh K, Lin SC. Association between serum ferritin and glaucoma in the South Korean population. JAMA Ophthalmol. 2014;132: 1414–1420. 10.1001/jamaophthalmol.2014.2876 25171442

[16] Ghosh. Comparison of anthropometric, metabolic and dietary fatty acids profiles in lean and obese dyslipidaemic Asian Indian male subjects. Eur J Clin Nutr. 2007;61: 412–419. 17006446 10.1038/sj.ejcn.1602534

[17] Levey AS, Stevens LA, Schmid CH, Zhang YL, Castro AF 3rd, Feldman HI, et al A new equation to estimate glomerular filtration rate. Ann Intern Med. 2009;150: 604–612. 19414839 10.7326/0003-4819-150-9-200905050-00006 PMC2763564

[18] Kim EC, Han K, Jee D. Inverse relationship between high blood 25-hydroxyvitamin D and late stage of age-related macular degeneration in a representative Korean population. Invest Ophthalmol Vis Sci. 2014;55: 4823–4831. 10.1167/iovs.14-14763 25015360

[19] Smith W, Mitchell P, Leeder SR, Wang JJ. Plasma fibrinogen levels, other cardiovascular risk factors, and age-related maculopathy: the Blue Mountains Eye Study. Arch Ophthalmol. 1998;116: 583–587. 9596493 10.1001/archopht.116.5.583

[20] Friedman E. The role of the atherosclerotic process in the pathogenesis of age-related macular degeneration. Am J Ophthalmol. 2000;130:658–663. 11078846 10.1016/s0002-9394(00)00643-7

[21] Peeters A, Magliano DJ, Stevens J, Duncan BB, Klein R, Wong TY. Changes in abdominal obesity and age-related macular degeneration: the Atherosclerosis Risk in Communities Study. Arch Ophthalmol. 2008;126: 1554–1560. 10.1001/archopht.126.11.1554 19001224 PMC5774859

[22] González AS, Guerrero DB, Soto MB, Díaz SP, Martinez-Olmos M, Vidal O. Metabolic syndrome, insulin resistance and the inflammation markers C-reactive protein and ferritin. Eur J Clin Nutr. 2006;60: 802–809. 16493453 10.1038/sj.ejcn.1602384

[23] Sun L, Franco OH, Hu FB, Cai L, Yu Z, Li H, et al Ferritin concentrations, metabolic syndrome, and type 2 diabetes in middle-aged and elderly chinese. J Clin Endocrinol Metab. 2008;93: 4690–4696. 10.1210/jc.2008-1159 18796516

[24] Jehn M, Clark JM, Guallar E. Serum ferritin and risk of the metabolic syndrome in U.S. adults. Diabetes Care. 2004;27: 2422–2428. 15451911 10.2337/diacare.27.10.2422

[25] Koorts AM, Viljoen M. Ferritin and ferritin isoforms I: Structure-function relationships, synthesis, degradation and secretion. Arch Physiol Biochem. 2007;113: 30–54. 17522983 10.1080/13813450701318583

[26] Koorts AM, Viljoen M. Ferritin and ferritin isoforms II: protection against uncontrolled cellular proliferation, oxidative damage and inflammatory processes. Arch Physiol Biochem. 2007;113: 30–54. 17558604 10.1080/13813450701422575

[27] Klein R, Deng Y, Klein BE, Hyman L, Seddon J, Frank RN, et al Cardiovascular disease, its risk factors and treatment, and age-related macular degeneration: Women's Health Initiative Sight Exam ancillary study. Am J Ophthalmol. 2007;143: 473–483. 17317391 10.1016/j.ajo.2006.11.058 PMC2812860

[28] Friedman A, Arosio P, Finazzi D, Koziorowski D, Galazka-Friedman J. Ferritin as an important player in neurodegeneration. Parkinsonism Relat Disord. 2011;17:423–430. 10.1016/j.parkreldis.2011.03.016 21550835

[29] Hahn P, Milam AH, Dunaief JL. Maculas affected by age-related maular degeneration contain increased chelatable iron in the retinal pigment epithelium and Bruch’s membrane. Arch Ophthalmol. 2003; 121: 1099–1105. 12912686 10.1001/archopht.121.8.1099

[30] Moore C Jr, Ormseth M, Fuchs H. Causes and significance of markedly elevated serum ferritin levels in an academic medical center. J Clin Rheumatol. 2013;19:324–328. 10.1097/RHU.0b013e31829ce01f 23965472

[31] Mainous AG 3rd, Wells BJ, Everett CJ, Gill JM, King DE. Association of ferritin and lipids with C-reactive protein. Am J Cardiol. 2004;93: 559–562. 14996579 10.1016/j.amjcard.2003.11.018

